# Correlation of Clinical Symptoms With Nasal Endoscopy and Radiological Findings in the Diagnosis of Chronic Rhinosinusitis: A Prospective Observational Study

**DOI:** 10.7759/cureus.16575

**Published:** 2021-07-23

**Authors:** Vanitha Brindha Baba Caliaperoumal, Dharanya GS, Prabu Velayutham, Balasubramanian Krishnaswami, Krishna Kumar Rama Krishnan, Nishanth Savery

**Affiliations:** 1 Ear Nose Throat, Sri Venkateshwaraa Medical College Hospital and Research Centre, Puducherry, IND; 2 Radiology, Sri Venkateshwaraa Medical College Hospital and Research Centre, Puducherry, IND

**Keywords:** vas, lund-kennedy score, lund-mackay score, nose, paranasal sinuses, nasal obstruction

## Abstract

Introduction: In the diagnosis of chronic rhinosinusitis (CRS), computed tomography (CT) of the nose and paranasal sinuses (PNS) remains the gold standard investigation. Though the diagnostic nasal endoscopy (DNE) is an equally effective and easily available investigation for diagnosis of CRS, its reliability and clinical significance to that of patient’s symptoms have to be proven.

Objective: The purpose of this study is to determine the correlations between the symptom severity score, radiological score, and the endoscopic score in the diagnosis of CRS.

Methods: This prospective observational study included 70 patients with CRS. It was conducted in a tertiary care institute from January 2019 to June 2020. All patients were subjected to DNE and CT nose and PNS. Symptom score was assessed using Visual Analogue Scale (VAS) score of 0-10. DNE and CT scores were calculated using the Lund-Kennedy endoscopic scoring system and the Lund-Mackay CT scoring system, respectively. The correlation between these scores were done using Pearson’s correlation coefficient (p-value).

Results: The mean and standard deviation of the symptom score by VAS was 7 ± 1.7; the Lund-Kennedy score was 7.6 ± 2.3, and the Lund-Mackay score was 14.3 ± 6.5, respectively. The symptom score had moderate correlation with the Lund-Kennedy Score (r = 0.643, p < 0.001) and high degree of correlation with the Lund-Mackay Score (r = 0.835, p < 0.001). The Lund-Kennedy score had a positive correlation with The Lund-Mackay score.

Conclusion: DNE can be utilized as an early diagnostic tool in the clinical evaluation of CRS and is equally effective as CT in diagnosing the same. At the same time, a CT scan can be done in patients with positive symptoms and can be reserved as a second-level investigation for those patients with negative endoscopic findings but who become symptomatic on follow-up.

## Introduction

Chronic rhinosinusitis (CRS) is a heterogeneous group of disorders characterized by chronic inflammation of the nose and paranasal sinuses (PNS). There is a wide geographical variation in the prevalence of CRS as it affects 5% to 12% of the general population [1]. It remains a common cause of morbidity, social embarrassment, impaired performance at school or workplace, and in addition to physical discomfort, it also causes a substantial economic burden to the patient in terms of missed workdays due to the physician or hospital visits [2]. A definitive diagnosis and timely intervention can reduce the morbidity of CRS.

CRS manifests itself in a varied way ranging from inflammatory thickening of sino-nasal mucosa to gross polyp formation. Based on endoscopic findings, it can be broadly classified as chronic rhino-sinusitis with nasal polyps and CRS without nasal polyps as per European Position Paper on Rhinosinusitis and Nasal Polyps (EPOS) 2012 guidelines [3].

For reaching towards a proper diagnosis and management of CRS, in 2007, new guidelines for rhinosinusitis from a multidisciplinary panel commissioned by the American Academy of Otolaryngology-Head and Neck surgery were published. The guidelines state that patients with 12 weeks or longer of two or more of the following signs and symptoms: mucopurulent drainage (anterior, posterior, or both); nasal obstruction (congestion); facial pain/pressure/fullness; or decreased sense of smell with additional information from the investigative modalities such as computed tomography (CT) scan of nose and PNS and diagnostic nasal endoscopy (DNE) can be used to diagnose, assess the severity of disease and plan the definitive line of management [4].

CT scan provides the ability to accurately assess these areas for evidence of localized disease or for anatomical defects that compromise ventilation and mucociliary clearance. This allows the surgeon to individualize their surgical approach according to the extent and location of the disease studied on a CT scan of the nose and PNS. While CT delineates the extent of the disease, defines any anatomical variants, and the relationship of the sinuses with the critical surrounding structures, the nasal endoscopy is inexpensive, easily incorporated into the routine examination, and helps in monitoring the progress of sinus disease. Most authors state a significant correlation between DNE and CT scan findings of the nose and PNS [5]. Hence, in recent times, both DNE and CT scans of the nose and PNS have revolutionized the understanding and management of CRS.

The purpose of this study was to determine the correlation between the symptom severity score, radiological score, and the endoscopic score in the diagnosis of CRS.

## Materials and methods

This prospective observational study was conducted from January 2019 to June 2020 at a tertiary care institute in South India. Informed consent was taken from all the patients who participated in the study. Approval from the Scientific Research Committee and Institute Ethics Committee was obtained. All provisions of the Declaration of Helsinki were followed. Patients above 18 years of age with signs and symptoms of CRS for more than three months duration and fulfilling the diagnostic criteria of CRS by the American Academy of Otolaryngology-Head and Neck surgery were included in our study. A convenient sampling method was used to recruit the participants of the study. Patients who were previously operated on, patients with facial anomalies, and pregnant patients were excluded from the study. Based on the correlation between clinical symptoms, endoscopy, and CT findings in the study by Pokharel et al., with 95% confidence and keeping α as 0.05, β as 0.2, and p-value <0.05 as significant, the sample size was calculated to be 70 [5].

**Figure 1:**
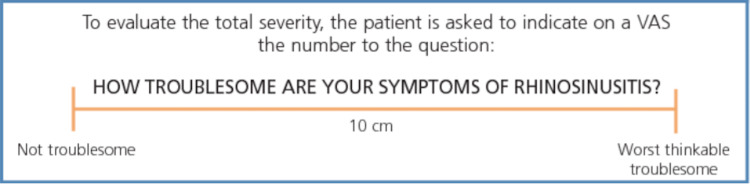
Visual Analogue Scale for assessment of symptom severity in patients with chronic rhinosinusitis.

Patients with CRS fulfilling the inclusion and exclusion criteria were enrolled in this study. Comprehensive clinical history was taken and detailed clinical examination was done on all patients. The symptom severity score was documented according to the visual analog method [6]. The intensity of every symptom was assessed on the Visual Analogue Scale (VAS) from 0 to 10, with scores 0 to 3 as mild, 4 to 7 as moderate, and 8 to 10 as severe symptoms, adapted from the ECOS primary care guidelines for the diagnosis and management of rhinosinusitis and nasal polyps (Figure 1) [7]. Patients were subjected to DNE and CT scans of the nose and PNS, and the findings were documented. 

DNE was performed after packing the nasal cavity with cotton pledgets soaked in 4% Lignocaine with 1:200,000 adrenaline for 7 to 10 minutes. The endoscopy was performed using a 4 mm 0-degree rigid endoscope by the same surgeon for all the patients to avoid inter-observer bias. The presence or absence of nasal polyps, edema, and discharge was noted, and the DNE score was derived according to the Lund-Kennedy endoscopic scoring system (Table 1) [8].

**Table 1:** The Lund-Kennedy endoscopic scoring method. OMC: Osteomeatal complex.

Right/left	Score 0	Score 1	Score 2
Polyps	Absent	In the middle meatus	Beyond the middle meatus
Discharge	Absent	Thin	Thick/purulent
Edema	Absent	Mild	Blocking the OMC

The nasal endoscopy was done systematically. It comprised of three passes, namely, first, second, and third. The first pass involved sliding the endoscope along the floor of the nasal cavities from an anterior to posterior direction. During this pass, the structures examined include the floor of the nose, inferior turbinate, and meatus, the nasopharynx, eustachian tube orifice, torus tubaris, and fossa of Rosenmuller. In the second pass, the scope is directed along the floor up to the posterior choana. It is then moved upwards, medial to the middle turbinate along the roof of the posterior choana and the anterior surface of the sphenoid. Structures visualized are superior turbinate, superior meatus, sphenoethmoidal recess, and the sphenoid ostium. The third pass involves studying the middle meatus area. The structures noted are the relation of the middle turbinate to the lateral wall, the uncinate process, accessory Ostia if any, concha bullosa, bulla ethmoidalis, and any other abnormalities.

The diagnostic evidence of CRS defined by the Lund-Kennedy endoscopic score ≥2 is significant. The patients diagnosed with CRS underwent a non-contrast CT scan of the nose and PNS. CT scan radiation exposure is nearly 185 times over that recorded for plain X-rays; hence, sensitive areas like eyes were covered properly and the patient was positioned properly. Mucosal thickening was studied systematically in the following area: the maxillary sinus, anterior ethmoids, posterior ethmoids, sphenoid sinus, frontal sinus, and the osteo-meatal complex; the findings were scored using the Lund-Mackay CT scoring system (Table 2) [9].

**Table 2:** The Lund-Mackay CT staging system for sinusitis based on CT scan findings.

Sinus	Score = 0	Score = 1	Score = 2
Maxillary	No abnormalities	Partial opacification	Total opacification
Anterior ethmoids	No abnormalities	Partial opacification	Total opacification
Posterior ethmoids	No abnormalities	Partial opacification	Total opacification
Sphenoid	No abnormalities	Partial opacification	Total opacification
Frontal	No abnormalities	Partial opacification	Total opacification
Osteomeatalcomplex	Not occluded	-	Occluded

According to the Lund-Mackay CT scoring, the score greater than or equal to 4 is the diagnostic value. Data collection was done in multiple stages for each patient. The demographic details, patient profile, relevant history, and examination findings were documented using a proforma. Symptom score was done with VAS, DNE findings were documented using the Lund-Kennedy endoscopic scoring system, and CT scan of nose and PNS were documented using the Lund-Mackay CT scoring system. The correlation between the symptom score, DNE score, and CT score were done using Pearson’s correlation coefficient (p-value) using the Statistical Package for Social Sciences (SPSS Software version 23, IBM Corp., Armonk, NY).

## Results

Among the 70 participants who were included in the study, most of the patients were between the age group of 21 to 60 years with the mean age group of the study population being 42.6±13.45 years. In the study population, 38 (54.3%) were female patients, and the remaining 32 patients were male (45.7%). Data were collected from all the 70 patients included in the study and there were no dropouts. Using the VAS, the severity of the symptoms was graded as mild, moderate, and severe of which four (5.7%) patients had mild symptom score, 36 (51.4%) patients had moderate symptom score, and 30 (42.9%) patients had severe symptom score (Table 3).

**Table 3:** Symptom severity grading using Visual Analogue Scale in patients with CRS. CRS: chronic rhinosinusitis, SD: standard deviation.

S.No.	Grading of symptoms	Number of patients (n=70) %
1.	Mild	4 (5.7%)
2.	Moderate	36 (51.4%)
3.	Severe	30 (42.9%)
Mean score (SD)		7 (1.7)

On performing DNE, 36 (51.4%) of them had polyps in the right nasal cavity and 44 (62.9%) of them had polyps in the left nasal cavity. Nasal cavity oedema was observed in 65 (92.9%) patients on both sides. Nasal cavity secretions were observed in 69 (98.6%) patients on the right nasal cavity and 68 (97.1%) patients on the left nasal cavity (Table 4).

**Table 4:** Diagnostic nasal endoscopy findings in right and left nasal cavity in patients with chronic rhinosinusitis using the Lund-Kennedy Endoscopic Scoring System. SD: standard deviation.

Characteristics		Nasal endoscopic finding (n = 70)	
		Right	Left
		N (%)	N (%)
Nasal cavity polyp score			
	No polyp	34 (48.58)	26 (37.14)
	Restricted to the middle meatus	11 (15.71)	9 (12.86)
	Below middle meatus	25 (35.71)	35 (50.00)
Nasal cavity oedema score			
	None	5 (7.1)	5 (7.1)
	Moderate	34 (48.6)	23 (32.9)
	Severe	31 (44.3)	42 (60)
Nasal cavity secretions score			
	None	1 (1.4)	2 (2.9)
	Clear and thin	43 (61.4)	43 (61.4)
	Thick and/or mucopurulent	26 (37.1)	25 (35.7)
Mean score (SD)			7.6 (2.3)

For CT findings, the Lund-Mackay CT scoring system was applied which revealed anterior ethmoids were more involved, followed by maxillary sinus, osteomeatal complex, posterior ethmoids, frontal sinus, and sphenoid sinus, respectively. In cases where anterior ethmoids were involved, 19 (27%) and 21 (30%) of patients showed partial opacification in the right and left nasal cavity, whereas 43 (61%) and 45 (64%) of patients showed complete opacification in the right and left nasal cavity, respectively (Table 5).

**Table 5:** Findings in CT scan of paranasal sinuses in patients with CRS (based on the Lund-Mackay CT Score). CT: computed tomography, CRS: chronic rhinosinusitis, SD: standard deviation.

S. No.	Sinuses	CT scan findings (n=70)	
		Right	Left
		n (%)	n (%)
1	Maxillary sinus		
	No opacification	8 (11.4)	7 (10)
	Partial opacification	15 (21.4)	14 (20)
	Complete opacification	47 (67.2)	49 (70)
2	Anterior ethmoids		
	No opacification	8 (11.4)	4 (5.7)
	Partial opacification	19 (27.2)	21 (30)
	Complete opacification	43 (61.4)	45 (64.3)
3	Posterior ethmoids		
	No opacification	19 (27.2)	16 (22.9)
	Partial opacification	15 (21.4)	22 (31.4)
	Complete opacification	36 (51.4)	32 (45.7)
4	Sphenoid sinus		
	No opacification	36 (51.4)	38 (54.3)
	Partial opacification	19 (27.2)	14 (20)
	Complete opacification	15 (21.4)	18 (25.7)
5	Frontal sinus		
	No opacification	32 (45.7)	30 (42.9)
	Partial opacification	15 (21.4)	22 (31.4)
	Complete opacification	23 (32.9)	18 (25.7)
6	Osteomeatal complex		
	No opacification	30 (42.8)	24 (34.3)
	Total opacification	40 (57.2)	46 (65.7)
Mean score (SD)			14.3 (6.5)

The mean and standard deviation of symptom score by VAS was found to be 7 ± 1.7; the Lund-Kennedy endoscopy score was found to be 7.6 ± 2.3, and the Lund-Mackay CT score was found to be 14.3 ± 6.5.

The correlation between the symptom score, the Lund-Kennedy endoscopic score and the Lund-Mackay CT scores were calculated by using Pearson’s correlation coefficient “r” (r<0.19 = slight, almost no relationship; 0.20-0.39 = low correlation; 0.40-0.69 = moderate correlation; 0.70-0.89 = high correlation; 0.90-1.00 = very high correlation). The symptom score had a moderate correlation with the Lund-Kennedy Endoscopic Score and was statistically significant (r=0.643, p<0.001), whereas the symptom score had a high degree of correlation with the Lund-Mackay CT Score and was statistically significant (r=0.835, p<0.001). The Lund-Kennedy endoscopic score had a positive correlation with the Lund-Mackay CT score (Table 6).

**Table 6:** Correlations between Symptom Score, the Lund-Kennedy endoscopic score, and the Lund-Mackay CT score **Correlation is significant at the 0.01 level (two-tailed). CT: computed tomography.

Correlations		Symptom score	The Lund-Kennedy endoscopic score	The Lund-Mackay CT score
Symptom score	Pearson correlation	1	0.644^**^	0.835^**^
	Sig. (two-tailed)		0.001	0.001
	N	70	70	70
The Lund-Kennedy endoscopic score	Pearson correlation	0.643^**^	1	0.722^**^
	Sig. (two-tailed)	0.001		0.001
	N	70	70	70
The Lund Mackay CT score	Pearson correlation	0.835^**^	0.755^**^	1
	Sig. (two-tailed)	0.001	0.001	
	N	70	70	70

Based on the study results, the symptom score based on the VAS had a positive correlation with the Lund-Kennedy endoscopic score and the Lund-Mackay CT score, and also, there is a positive correlation between the Lund-Kennedy endoscopic score and the Lund-Mackay CT score.

## Discussion

Chronic rhinosinusitis is a common disease that is increasing worldwide. Though the diagnosis of CRS is clinical and symptom-based, the American Academy of Otolaryngology task force on Rhinosinusitis recommends nasal endoscopy and CT scan of nose and PNS for accurate diagnosis and effective management of the disease [4]. In our study, the findings of nasal endoscopy and CT scans were assessed using the Lund-Kennedy endoscopic scoring system and the Lund-Mackay CT scoring system. Many authors have researched to seek out the correlation between subjective symptoms and objective disease parameters in CRS patients to get a far less complicated, faster, cheaper, and reliable way to make the right diagnosis and choose the right and timely treatment; however, the results were controversial. The present study was undertaken to find the association between subjective symptom severity with nasal endoscopy and CT findings in patients with CRS.

In the present study, 46% of patients were male, and 54% were female, similar to the study by Clifton and Jones on gender distributions where 55% of the study population was females [10]. In the study by Park et al. on CRS, the male-to-female ratio was 1:1.35 [11]. 

Chronic rhinosinusitis is much more prevalent in females due to differences in anatomic size, tobacco susceptibility, and hormonal factors, which have been speculated to increase the overall susceptibility to CRS in women compared with men. Women may be more susceptible to obstruction and subsequent infection due to smaller sinus Ostia [12,13].

In this study, we observed that the most predominant symptoms are nasal obstruction and nasal discharge in almost all the enrolled cases. Nayak et al. and Deosthale et al. in their studies found similar results, as patients with CRS had a nasal obstruction and nasal discharge as the predominant symptoms [14,15]. 

The objective evaluation of CRS is based on the CT appearance and endoscopic findings. The CT scores are derived from the Lund-Mackay CT scoring system that attributes points based on sinus mucosal disease, opacification, and obstruction. Bhattacharya used the Lund-Mackay CT scoring system to grade the severity of CRS, based on whether the sinus is clear, partially opacified, or totally opacified [16]. 

The average Lund-Mackay CT score in our study was 14.3. In the study by Bhattacharya on paediatric patients [16], the mean Lund score was 10.4 among 66 cases and it was 2.8 among 192 controls. Singh et al. in their study on CRS patients undergoing FESS identified that patients with CT scores of more than 13.1 had better clinical outcomes after the surgical procedure [17]. 

CT scan has a high sensitivity to identify mucosal inflammation in the nose and PNS and hence can overestimate incidental mucosal findings for true sinus disease. For instance, the presence of polypoidal mucosa makes the appearance of the CT scans to be severe, with a corresponding higher score assigned [18].

Clifton et al., based on their findings, advised not to operate on asymptomatic patients based on CT score alone as even in asymptomatic patients, CT scan shows abnormalities in 30% of cases [10]. Hence, it is always preferable to correlate symptoms and CT findings and then decide on surgical management.

Our study did not find any significant correlation between individual symptoms such as nasal obstruction (r=0.25) and nasal discharge (r=0.183) of CRS with the CT Score. Holbrook et al. found no correlation between actual facial pain with the corresponding sinus opacification on CT scans [19]. The study conducted by Bhattacharyya et al. and Bradley and Kountakis also compared sinonasal symptoms with CT findings and found no significant association between them [20,21]. Kenny et al. showed a mild positive correlation between total symptoms and the Lund- Mackay CT scores, except between facial pressure/pain and CT finding [22]. Since more emphasis was given to symptomatic diagnosis, there were significant discrepancies in the results between various studies conducted in the past. As the severity score of the symptoms increased (moderate and severe), the correlation of symptom score with the Lund - Mackay CT scores increased significantly.

In our study, the symptom score had a moderate correlation with the Lund-Kennedy Endoscopic score (r=0.643, p<0.001), which is similar to the study done by Tomassen et al., where it was found that symptom-based CRS was statistically associated with positive endoscopy findings [23].

In our study, the endoscopy score had a good correlation with CT finding (r=0.835, p=0.001) which is similar to a study conducted by Deosthale et al. who showed a correlation coefficient of 0.881 (p-value <0.0001) between the Lund-Mackay CT Score and the Lund-Kennedy endoscopy score [15]. Rosbe et al. recorded that patients with positive findings on endoscopy had positive CT scan reports, too [24]. Similarly, Deosthale et al. and Buljcik-Cupic and Savović also showed a high degree of agreement between endoscopy and CT scanning for examining nasal cavities with kappa greater than 0.8 and 0.7 [15,25]. The results showed a strong correlation between endoscopy and CT findings in CRS.

According to Bhattacharyya and Lee, when endoscopy findings were combined with symptom scores, it significantly increased the diagnostic value compared with CT scans [26]. Ferguson et al. concluded that endoscopy has high specificity but low sensitivity; hence, it can only be used for diagnosing patients with CRS but not for ruling it out [27]. Deepthi et al., in their study, found a positive correlation between subjective symptom severity and objective endoscopic and radiologic finding [28].

According to Kasapoglu et al., CT and nasal endoscopy are supplementary to each other in the pre-operative evaluation of patients with chronic sinusitis [29]. According to Pullarat et al., in CRS, DNE can prove to be a higher diagnostic modality when compared to CT scan in assessing the nature of the secretions (mucoid, mucopurulent, purulent, blood-stained), condition of the nasal mucosa (pale, congested, and presence or absence of polyps) [30]. An added advantage of DNE is that, in pathological nasal mass, histopathology is crucial for its diagnosis, where DNE can help take a precise biopsy to establish the benign/malignant nature of the nasal mass. Endoscopic-directed procedures have a high degree of accuracy because of vision-controlled and incomparable guidance in treating nasal and nasopharyngeal pathologies and in skull base surgeries (olfactory neuroblastomas, pituitary adenomas).

Even though CT scanning of nose and PNS is considered to be the gold standard in the diagnosis of CRS, it is not routinely advised in our setup due to the cost factor and radiation exposure. Hence, it can be done in patients with positive symptoms and can be reserved as a second-level investigation for those patients with negative endoscopic findings but who become symptomatic on follow-up. In cases where it is difficult to navigate the endoscope beyond a certain point, either due to severe anatomical abnormalities like a gross deviation of the nasal septum, paradoxical middle turbinate, or a concha bullosa, a CT scan can definitely be helpful.

## Conclusions

Diagnostic nasal endoscopy can be utilized as an early diagnostic tool in the clinical evaluation of CRS and is equally effective as CT in diagnosing the same. At the same time, CT can be done in patients with positive symptoms and can be reserved as a second-level investigation for those patients with negative endoscopic findings but who become symptomatic on follow-up.
